# The production process of chepa shutki: A traditional Bangladeshi fermented fish product

**DOI:** 10.1016/j.heliyon.2025.e41972

**Published:** 2025-01-16

**Authors:** Jasmin Ara Farhana, Sijmen E. Schoustra, Ferdous Ahamed, Bas Zwaan, Oscar van Mastrigt

**Affiliations:** a Patuakhali Science and Technology University, Bangladesh; b Food Microbiology, Wageningen University & Research, P.O. Box 17, 6700 AA, Wageningen, The Netherlands; c Laboratory of Genetics, Wageningen University & Research, P.O. Box 17, 6700 AA, Wageningen, The Netherlands; d Department of Food Science and Nutrition, University of Zambia, Lusaka, Zambia

**Keywords:** Fermentation, Spontaneous, Indigenous, Motka, Anaerobic, Food security

## Abstract

Chepa shutki is a sticky, solid, fermented, salt-free product from Bangladesh prepared from small-sized fish by local producers. It can be consumed as a chutney or sauce-like recipe or shutki curry with vegetables as a side dish for rice or bread. Chepa shutki is manufactured according to a small-scale traditional technique relying on spontaneous fermentation. Although chepa shutki is an important part of the human diet in Bangladesh, providing flavour as well as essential nutrients (especially protein and bone minerals), research on variations in production of chepa shutki has been limited. The aim of this study was to determine the variations in processing methods of chepa shutki and identify production process parameters. A survey was carried out with respondents using structured questionnaires and observations on current processing practices and the equipment used. In Bangladesh, chepa shutki is produced from different small fish (*Puntius, Setipinna* and *Otolithoides*) referred to as punti, phaisha and puma type, respectively. Among these, the punti type is produced the most and puma type the least. After washing, sun drying, soaking and overnight storage, fermentation takes place in airtight earthen pots (locally called motka) whose micropores are blocked with fish oil to ensure anaerobic conditions. The fermentation duration varies from three to twelve months. Types of fish, types of fermentation container (old/new) and fermentation time were identified as vital process parameters. This information is crucial for further studies to understand how processing variations affect product properties and to develop a standard processing method to produce consistently high quality chepa shutki with good preservation properties.

## 1 Introduction

Traditional fermented foods have a long-standing relationship with the region, season and availability. They are loved, enjoyed and are part of a tradition in several regions of the world [1]. They are often not commercially produced on an industrial scale, but rather in local cottage industries and often are only available locally [2]. Traditional fermentation increases food security by preserving raw materials as fermented products to combat the scarcity of food that could prevail in the months after the peak season. Moreover, fermentation can enhance the flavour and nutritional value of food products. A wide range of fermented foods and beverages are produced from fresh foods, such as milk, cereals, fruits, beans, vegetables, meat and fish. Each country has developed unique ways of preserving food, especially for meat and fish [3].

The production of traditional fermented foods is driven by microorganisms present in the raw food material or fermentation vessels (spontaneous fermentation) via traditional methods that are employed by the artisanal processor in homes [4]. The processes have been improved mostly by trial and error, based on the observations of the practitioners without any scientific support [5], resulting in a large variation of existing processing methods. Combined with geographical availability of raw materials, traditional fermented foods have a high degree of variability in microbial populations and unique product characteristics [1].

Spontaneously fermented fish products play an important role in the gastronomy and contribute significantly to the food and nutrition security of the rural population by increasing the availability and affordability of protein-rich food products. Fermented fish products are nutritious because fish is rich in protein and fermentation breaks the proteins down into more easily digestible peptides enhancing the nutritional value [6]. Moreover, fish bones are rich in minerals such as calcium, zinc and phosphorus and become softer and more munchable after being fermented [6]. Fermentation also increases the absorption of nutrients due to the smaller particle size compared to fresh fish [6] because hydrolysis of food proteins by microbial enzymes yield simpler compounds such as peptides, amino acids and other nitrogenous substances [7]. It can reduce anti-nutritional factors [8,9] and provide antioxidant activity [10,11]. Furthermore, traditional fermentation of fish improves the flavour, appearance and texture and fermented fish can provide additional functions to people's health, including appetite, digestion and beneficial microorganisms [3,12]. However, one of the most important benefits of fermented fish is the increased shelf life of fish products.

Due to seasonal variation in supply of perishable, small, commercially unimportant freshwater and salt water fish, fish is preserved to use as food during the dry season in several Southeast Asian countries such as Thailand, Indonesia, Philippines, Malaysia, Vietnam, Bangladesh and northeastern parts of India. Fish can be preserved by canning, freezing, drying, salting or fermentation [13]. Of these methods, fermentation is one of the oldest and most preferred practices and fermented fish products are embedded in the culture of the local communities and valued for their nutritional value. Fermentation of fish is a complex decomposition process, in which the metabolic activity of microorganisms and their enzymes degrade proteins into peptides and amino acids and further to a range of flavour compounds. The microbes in this process come from fish itself, the salt used as well as the equipment [14]. Varieties of traditional fermented fish products result from the different fish species used as the primary raw material, salt concentrations, storage containers and storage temperatures [15].

Over the past decade, extensive research related to fermented fish products were conducted in various Asian countries such as on bagoong [16], patis [16], balao-balao [17] and burong bangus [18] in Philipines; bakasang [19], bekasam [20], peda [21], ronto [22] and terasi [14] in Indonesia; belacan [23], budu [24] and pekasam [25] in Malaysia; chouguiyu [26] and yu-lu [27] in China; gajami-sikhae [28] and jeotgal [3] in Korea; ishiru [19,29], narezushi [30] and shottsuru [29] in Japan; kapi [31], nam-pla [19] and pla-ra [32] in Thailand; nga-pi [33] and chepa shutki [34] in Bangladesh; ngari, hentak, tungtap [35] and shidal [36] in India and nuoc-mam [37] in Vietnam. These studies demonstrate that fermented fishery products are commonly sold and consumed in areas where they are produced.

Chepa shutki is one of Bangladesh's signatory traditional fermented fish products, whose production is concentrated near the haor region. It is usually made using various small fishes, including salt-water and fresh-water fish [10]. Although it is widely consumed especially in some parts of Mymensingh, Netrokona, Brahmanbaria and Kishoreganj district of Bangladesh, it is popular among all Bangladeshi. The product is appreciated for its firm but tender texture, strong odour and desirable taste [38]. There are several types of chepa shutki available, the names of which are derived from the type of fish used. Punti chepa shutki is the most popular chepa shutki, especially in Mymensingh. The general traditional procedure for chepa shutki production involves spontaneous fermentation by naturally present microorganisms. After washing, drying, soaking and overnight storage, the fish is stored in a tightly sealed earthenware jar and fermented for 3–12 months at ambient temperature under anaerobic conditions [39]. It is considered that longer fermentation makes the product taste better.

However, because the fish is fermented traditionally, undesirable microorganisms sometimes interfere causing loss of this valued food product. To prevent food loss and improve the quality of chepa shutki, it is important to know the existing variations in processing and how these variations impact the quality of product, such as the organoleptic properties, nutritional value and safety. Although many studies have determined the nutrient profile of chepa shutki, information on variations in processing method of chepa shutki and their effect on the product quality is lacking [36,[40], [41], [42], [43]].

To fill this gap, the aim of this study was to map and document variations in processing steps of chepa shutki at eleven upazilas, administrative divisions in Bangladesh that function as sub-units of a district, of Bangladesh where chepa shutki is produced traditionally. This study used a survey approach (interviews were conducted with producers using a structured questionnaire) to document and understand how producers made their product and identify the key processing steps.

## 2 Materials and methods

### 2.1 Study area and sampling

**Fig. 1:**
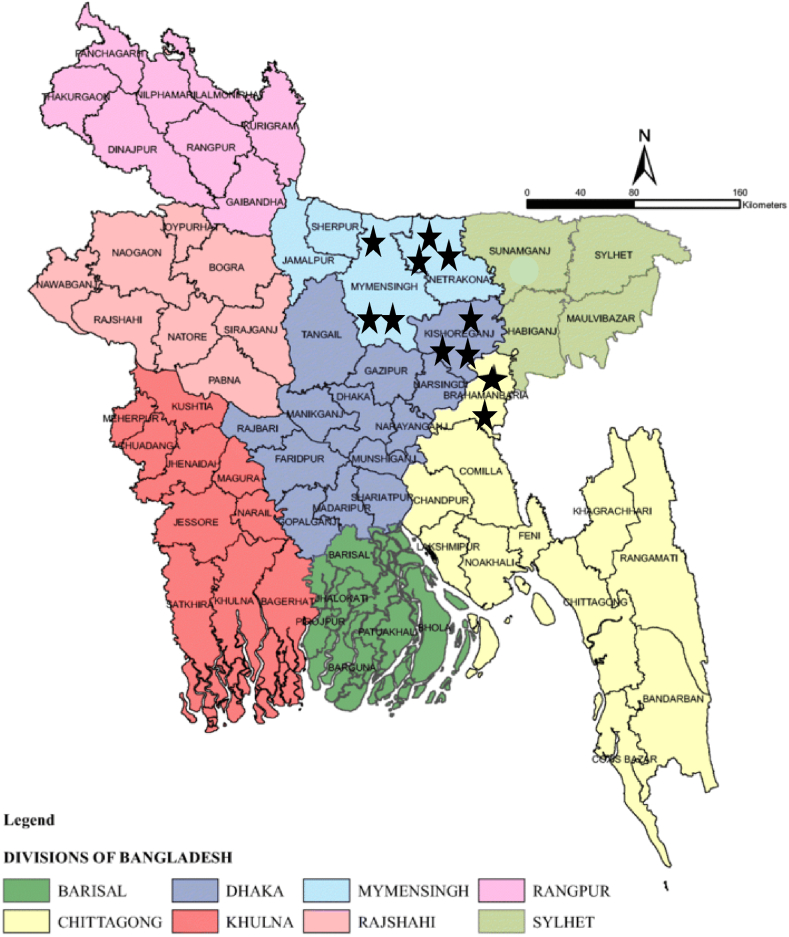
Map of Bangladesh showing the sampling sites in Mymensingh, Netrokona, Kishoregonj and Brahmanbaria (black stars).

A cross sectional study was carried out to collect samples of chepa shutki from 120 producers in eleven upazilas of Bangladesh between December 2022 and January 2023 (Fig. 1). The upazilas and producers of chepa shutki were selected based on information of chepa shutki producers of Bangladesh provided by the district fishery officer. Our target was to find at least five producers of a specific type of chepa shutki from each upazila.

### 2.2 Data collection

The respondents that provided samples were interviewed during the survey. Structured questionnaires were administered in one-to-one interviews in local languages that were most familiar to the producers. Demographic information of the respondents is given in Table 1. At least 5 questionnaires were administered per upazila and the following information was recorded for all samples collected: sampling location (upazila and district), production method, age of chepa shutki, type of fermentation containers used, type of fish used (*Puntius*, *Setipinna* or *Otolithoides*), duration of water soaking and socio-demographic information of the producers. Additionally, the production practices and the equipment used were recorded.

**Table 1:** Demographic information of the respondents.

Parameter & Descriptor	Percentage
Marital status	
Unmarried	13
Married	87
Household size	
3-4	29
5-6	49
≥7	22
Age (years)	
<20	3
20-29	22
30-39	28
40-49	18
50-59	18
>59	12
Religion	
Muslim	33
Hindu	67
Education level (class)	
No education	9
1-5	46
6-10	31
>10	14

Focus group discussions (FGDs) were conducted with producers in groups of 5–7 people and 1–7 FGDs were carried out per upazila depending on the number of respondents available.

Semi-structured interviews were carried out with key informants, including chairpersons of fish processing centers and local chiefs as well as upazila fishery officers, using a set of questions (Checklist) on the production process of chepa shutki and its application.

## 3 Results

### 3.1 Chepa shutki production steps

**Fig. 2:**
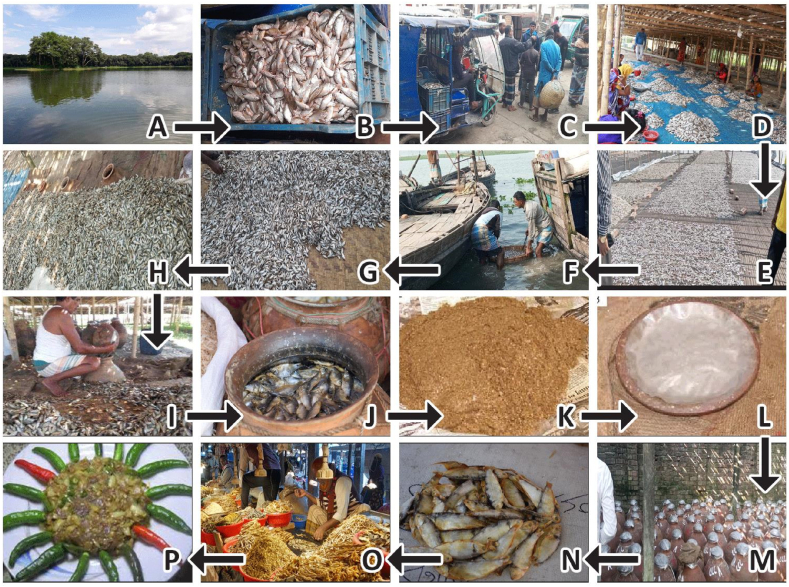
Processing steps of chepa shutki production (A–P). A: a beel proving an excellent habitat for weed fish such as Puntius. B: Collected Puntius fish. C: Transportation of fish to fish processing centre. D: Dressing, gutting and sorting of fish by female workers. E: Drying of fish. F: Soaking dried fish in river. G: Spreading soaked fish on Bamboo mat for overnight storage. H: Gathering dried, soaked and overnight stored fish for filling into motka. I: Filling of motka with fish. J: Filled motka. K: Cover paste of ground dried fish used to fill mouth of motka. L: The cover paste is covered with a layer of wet clay and sealed with polythene. M: Storage of motkas for 3–12 months. N: Chepa shutki. O: Market selling chepa shutki. P: Dish containing chepa shutki.

**Fig. 3:**
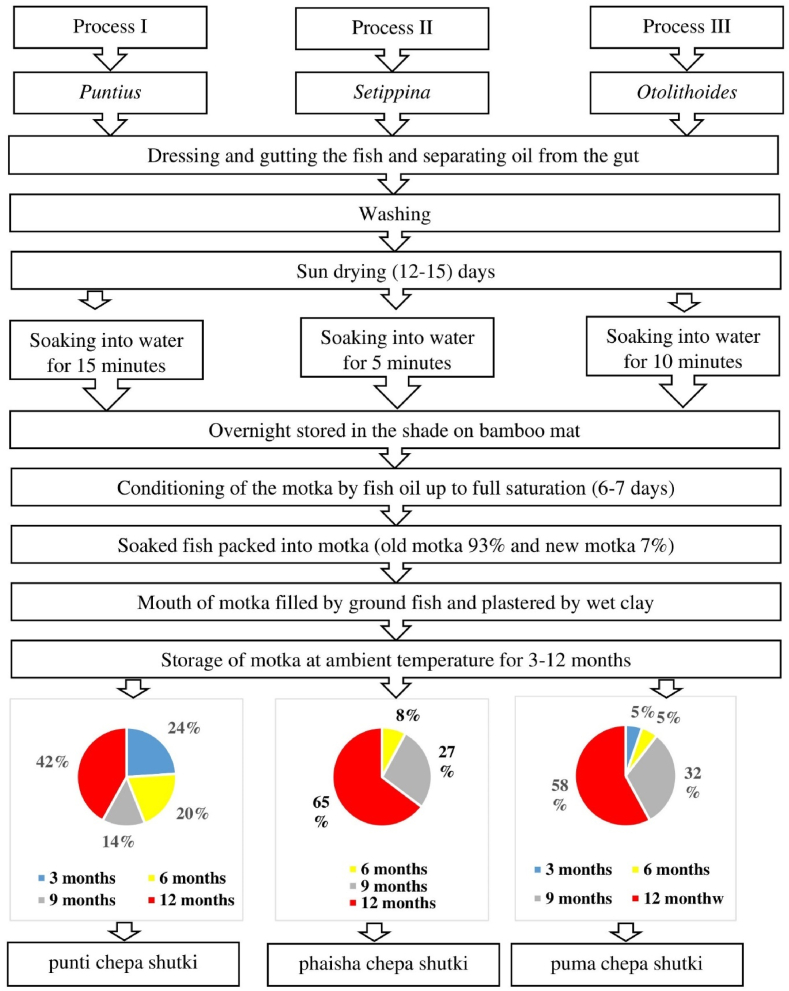
Flow diagram of production of three types of chepa shutki.

To determine the traditional production practices of chepa shutki, 120 different local producers of chepa shutki from eleven upazilas have been visited and structured questionnaires have been administered (Table 2). Based on this survey, a general production process for chepa shutki has been constructed, which is illustrated in Fig. 2. Moreover, the survey revealed three distinct methods of chepa shutki production, each yielding a distinct product (Fig. 3).

**Table 2:** Number of chepa shutki samples collected from each sampling location.

District & Upazila	Number of samples collected per upazila			
	Punti chepa shutki	Phaisha chepa shutki	Puma chepa shutki	Total
Netrokona				
Sadar	8	9	–	17
Barhatta	7	8	–	15
Mohongonj	–	7	–	7
Mymensingh				
Sadar	10	–	–	10
Fulpur	5	–	–	5
Tarakanda	5	–	–	5
Brahmanbaria				
Sadar	–	7	–	7
Ashugonj	–	15	19	34
Kishoregonj				
Sadar	9	–	–	9
Kotiadi	6	–	–	6
Kuliarchar	–	5	–	5

### 3.2 Chepa shutki production methods

#### 3.2.1 Punti chepa shutki

**Fig. 4:**
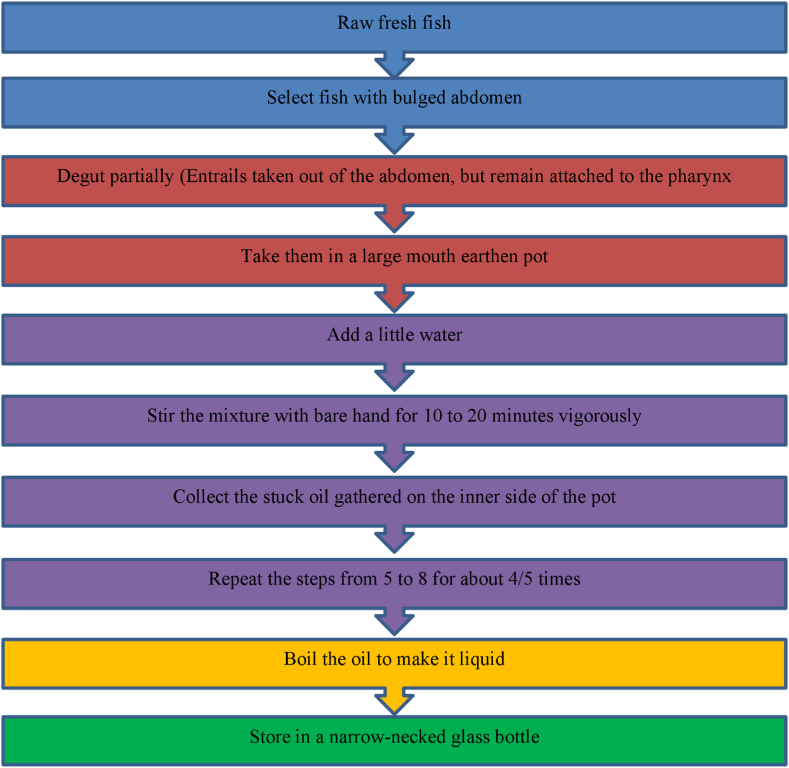
Indigenous fish oil extraction.

The typical production process of punti chepa shutki is illustrated in a flow diagram in Fig. 2, Fig. 3 (process I). The production of punti chepa shutki begins with fishing the small freshwater fish *Puntius,* of which the size varies from less than 5 cm–25 cm depending on species. Afterwards, the raw fish is transported from the fishing site to the fish processing center by pick up, van and boat in a container made of bamboo, steel or plastic. Then, the raw punti fish is dressed, gutted and oil is extracted from the entrails of the fish (Fig. 4).

After washing, raw fish is dried in the sun for about 12–15 days. The dried fish is soaked into river water for 15 min because its skin is very thick. Then the soaked fish are spread on a bamboo mattress and stored in the shade overnight. Water soaking and subsequent storage in the shade gives the fish a soft texture with a dry surface, but more importantly, these steps are critical in the process of chepa shutki to control the water activity and determine which microorganisms dominate the fermentation. Afterwards, the fish is ready for filling in the fermentation container.

Traditionally, motka's made from fine black soil in Savar upazila, Dhaka, are used for fermentation. To create anaerobic environments during fermentation, motka's are made impermeable to air using oil. Fish oil extracted from the same fish as the chepa shutki is smeared on both inner and outer walls of the motka to full saturation thereby blocking the micropores of the walls of the motka. The motka is subsequently dried for several days. A motka can be new or old. Though the price of an old motka is higher than the price of a new motka, an old motka (93 %) is preferred because it requires less oil to become fully saturated. Moreover, less time is required for the drying (2–5 days compared to 7–10 days). Before filling the motka, one-third of the motka's belly is buried in the ground to fix it in a vertical position to the ground. To avoid any contamination from the soil underneath, clean gunny bags are spread surrounding the motka. A layer of dried, soaked and overnight stored fish is spread into the motka with compaction by applying uniform pressure with bare hands and a wooden stick. Similar airtight packing of fish layers continues up to the mouth region of the motka.

Subsequently, the mouth of the motka is sealed with a 5.00–6.50 cm layer of cover paste, which is prepared by grinding dried fish. The cover paste is protected from flies and maggots by applying a thick layer of clay prepared from wet mud of fine soil. To ensure anaerobic condition inside the motka and get high-quality fermented fish, the clay layer is covered with a sheet of polythene and checked for about 2 weeks. Fresh wet mud is used to repair any visible cracks. The mud-sealed motka is then lifted from the buried position and left undisturbed in the shade to ferment spontaneously for 3–12 months at an average temperature of 28 °C.

#### 3.2.2 Phaisha chepa shutki

The production process of phaisha chepa shutki is similar to punti chepa shutki, but uses fish of the genus *Setipinna,* the hairfin anchovies, found in estuaries (Fig. 3, process II). Different species of *Setipinna* has different length that vary between 11 and 40 cm. Because the skin of *Setipinna* is thin, dried *Setipinna* fish are only soaked in water for 5 min.

#### 3.2.3 Puma chepa shutki

Fish from the genus *Otolithoides* are used to produce puma chepa shutki. These are marine ray-finned fishes available at estuarine systems. Due to variation in species, their size varies from 11 to 38 cm. Puma chepa shutki is produced in the same way as punti and phaisha chepa shutki except for the water soaking time of 10 min because the thickness of the skin of Puma fish is in between the thickness of the skin of Punti fish and Phaisha fish (Fig. 3, process III).

Comparison of the fermentation durations of the collected chepa shutki samples revealed that chepa shutki is mostly fermented for at least 9 months, in particular for phaisha and puma chepa shutki (>90 %) (Fig. 3). In contrast, short fermentation times (three months) are more prevalent for punti chepa shutki (24 %, 0 % and 5 %, for punti, phaisha and puma chepa shutki, respectively).

### 3.3 Chepa shutki processing characteristics

**Fig. 5:**
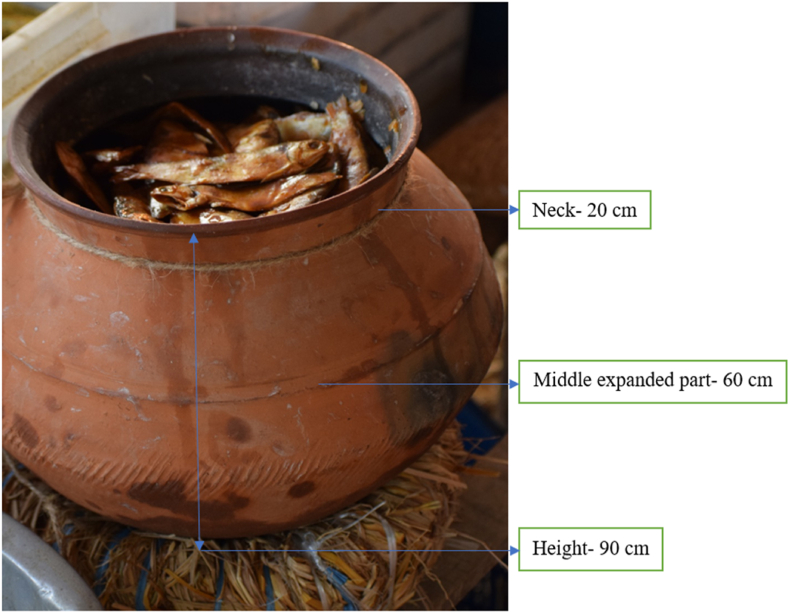
Motka used for the production of chepa shutki.

The quality of chepa shutki depends on its processing characteristics, such as fish species, source of used water, old or new motka etc. An overview of the processing characteristics is given in Table 3. Chepa shutki is produced mainly from *Puntius*, *Setipinna* and *Otolithoides* fish. River and beel water are used at the fish processing centre to wash the fish, to soak dried fish and to wash motka's for reuse. Oil smearing acts as a protective cover inside the motka to inhibit the action of putrefying bacteria. Old motka's need less oil to smear than new motka's (0.2–0.5 L and 1.5 L, respectively). One motka can be used to produce 36–42 kg of chepa shutki. A representative picture of a motka is given in Fig. 5. Of the three chepa shutki types, punti chepa shutki is the most expensive (i.e. 1300 taka per kg, about 10 euro/kg). The production of chepa shutki occurs from October to February and its sale is ongoing from January to yearlong. Besides national consumption, a portion of produced chepa shutki is exported to India.

**Table 3:** Description of variations in chepa shutki production practices including material, equipment, price and infrastructure.

Parameter	Descriptor
Mostly used fish species	*Puntius, Setipinna, Otolithoides*
Characteristics of processing centre	
Electricity	Present
Water source	River, pond and beel
Floor condition	Cemented
Building block of infrastructure	Tin, rope and bamboo
Motka	
Neck of motka	20 cm
Middle expanded part of motka	60 cm
Height of motka	90 cm
Weight of empty motka	14–18 kg
Weight of filled motka	50–60 kg
Oil needs to smear motka	
New motka	1.5 L
Old motka	0.2–0.5 L
Chepa shutki produced per motka	36–42 kg
Price of chepa shutki (per kg)	
Punti	1300 taka
Phaisha	700 taka
Puma	1000 taka
Chepa shutki production schedule	
Start	From October
Continue	October to February
Chepa shutki sale	
Start	From January
Continue	January to yearlong

### 3.4 Distribution of the chepa shutki production methods

**Fig. 6:**
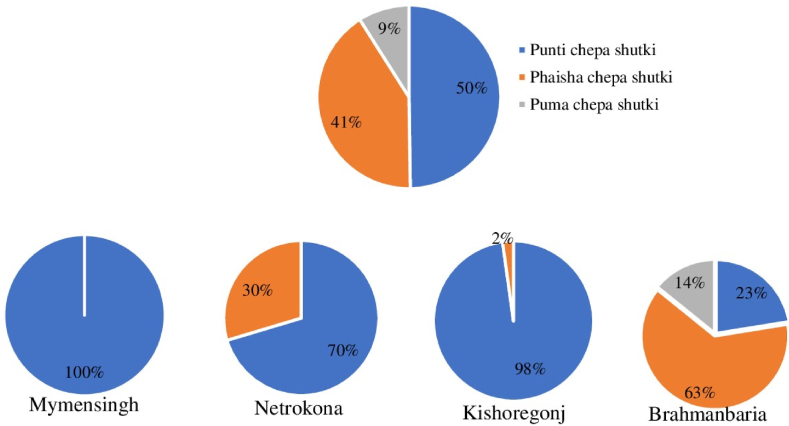
Distribution of chepa shutki types in Mymensingh, Netrokona, Brahmanbaria and Kishoreganj. The large pie chart shows the average distribution for the country whereas each small one shows the regional production of three types of chepa shutki.

Chepa shutki is mainly produced in the four investigated districts of Bangladesh. Of the three chepa shutki production methods, punti chepa shutki is produced most frequently in Bangladesh in the investigated districts (50 %), followed by phaisha chepa shutki (41 %) and puma chepa shutki (9 %) (Fig. 6). These production methods were not equally spread over the country but were dependent on the availability of the fish in the districts. Mymensingh district produces only punti chepa shutki whereas the other three districts produce phaisha chepa shutki together with punti chepa shutki to different extents. Moreover, puma chepa shutki is exclusively produced in Brahmanbaria. After production, the producers sell their motka to other parts of Bangladesh.

## 4 Discussion

This study aimed to map and document variations in chepa shutki processing methods in Bangladesh. This study shows that there are three different production methods of chepa shutki in Bangladesh. The most important variations were the types of fish, fermentation time and types of container (old or new).

Both freshwater *Puntius*, estuarine system *Setipinna* and marine *Otolithoides* can be used for chepa shutki production. As expected, which fish is used for chepa shutki production is mainly dependent on the local availability. Puma chepa shutki is produced only in Brahmanbaria district because *Otolithoides* is not available in the other districts, while *Setipinna* fish is available in Brahmanbaria, Netrokona, Kishoregonj and used in these districts to make chepa shutki. Finally, in Mymensingh only punti chepa shutki is produced because *Puntius* is widely available in this district. The unique flavour and availability of punti chepa shutki makes it the most popular chepa shutki among the three types of chepa shutki.

The fermentation time of chepa shutki production varies from 3 to 12 months. During fermentation, bacteria progressively digest and solubilize the fish solids (protein and lipids) into peptides, amino acids, fatty acids, indole, skatole among others, which are responsible for the strong characteristic flavour of chepa shutki [44]. Longer fermentation times increase the flavour and the availability of nutrients, especially protein and minerals such as calcium, phosphorus, magnesium and iron, reaching higher mineral levels than in similar Japanese processed fish [38]. Moreover, longer fermentation result in a softer texture of the chepa shutki. A wide variety of fermentation times has also been found in processes of other fermented fish products, affecting their product characteristics. For instance, the short fermentation period of 8–12 days of plaa-som [23] production is not long enough for the fermenting bacteria to soften and munch fish bones properly, resulting in a low availability of bone mineral. In contrast, patis is fermented for one to two years to properly digest the fish [45]. In case salt is added to the fish to inhibit spoilage bacteria and favour growth of salt-tolerant bacteria, such as during nuocmam production, longer fermentation times of 12–15 months are required to extensively liquefy the fish tissue [37].

The fermentation of chepa shutki takes place in earthenware jars, called motka. Old motka's are mostly used due to lower costs because less oil is needed to saturate them. Saturation of the motka with oil increases the product quality by lowering the air permeability, allowing a fermentation process in anaerobic conditions. Moreover, the oil minimizes desiccation through evaporation and seepage [46] and prevents water absorption from the fish after filling them in the motka. Finally, the oil adds flavour to chepa shutki [44]. Airtight earthenware pots are commonly used to ferment fish under anaerobic conditions, such as during production of nuoc-mam, budu [47], ngari, hentak and tungtap [48].

Like lanhouin, chepa shutki is produced without standardization of the production methods and hygiene [49]. While Prahok, a Cambodian staple food, is produced at industrial scale by following strict industry standards, all chepa shutki varieties are produced at small scale without underlining certain specific characteristics like colour and smell [23]. In most fermented fish products salt is added during fermentation to favour the growth of salt-tolerant microorganisms and steer microbial community, such as in sikhae (<7 %), jeotgal (20–30 %) [28], Yu-lu (30 %) [27]. In contrast, production of chepa shutki is free from salt addition. While consumption of high salt containing fish sauces might threaten consumers’ health, chepa shutki is safe for the consumption by patients having cardiovascular disease and sodium restricted diets [49]. In the production of plaa-som, suan yu, pekasam and narezushi, carbohydrate sources such as rice, millet, flour and sugar are added besides salt to the raw material [49]. These carbohydrate sources provide the microorganisms with a source of energy to accelerate the fermentation and impart a characteristics flavour to the end product. In contrast, all chepa shutki varieties are made without addition of carbohydrate sources and the breakdown of fish protein and lipids produce the characteristic flavour of chepa shutki.

## 5 Conclusion and outlook

Different chepa shutki processing variations exist, which likely impact the quality of the chepa shutki. The documentation and systematically mapping of these existing variations in processing was the main aim of the present study and now inspires further study on how these processing variations may affect specific product attributes. To date, the variations in processing steps of chepa shutki has not been formalized. To systematically assess these variations, this study used a survey approach. Following established scientific approaches for cross-sectional surveys, we conducted interviews with producers using a structured questionnaire taking care of sufficient numbers of respondents based on established scientific criteria. With this, we were able to document and understand variations in how producers made their product and identify the key processing steps.

This knowledge is crucial for further investigations on how processing variations affect the fermentation process and the product characteristics, which need to be rooted in existing processing variations. For instance, further investigations could now couple the processing variations to steering the microbial community compositions and the associated product quality characteristics such as aroma, taste, nutrition value, texture and safety. Systematic research on the processing variations and their impact on the quality will help to understand how processing variations affect the quality of traditional fermented fish products and to identify the critical steps. By implementing this knowledge, higher nutrient and flavour containing traditional fermented fish products can be developed, which will benefit producers throughout the world. Moreover, standardization of fish fermentation could play a vital role in the economy of a country by increasing food production, diversifying the economy, increasing employment opportunities, enhancing rural peoples’ livelihood as well as improving nutritional and health status of the consumers.

## CRediT authorship contribution statement

**Jasmin Ara Farhana:** Writing – original draft, Methodology, Formal analysis, Conceptualization. **Sijmen E. Schoustra:** Writing – review & editing, Supervision, Resources, Project administration, Funding acquisition, Conceptualization. **Ferdous Ahamed:** Writing – review & editing. **Bas Zwaan:** Writing – review & editing, Supervision. **Oscar van Mastrigt:** Writing – review & editing, Supervision.

## Data availability

The data and materials related to this study are available upon request.

## Ethics declarations

Ethical guidelines of Wageningen University and Research were followed in this study.

Permission to conduct the interviews for the purposes of this research was obtained by all respondents, who were fully informed about the purposes of this research and how their responses would be used.

## Funding

This work was supported through a WGS PhD fellowship from 10.13039/501100004890Wageningen University and Research, The Netherlands.

## Declaration of competing interest

The authors declare that they have no known competing financial interests or personal relationships that could have appeared to influence the work reported in this paper.
